# Raw Meat in the Diarrhœa of Children

**Published:** 1859-09

**Authors:** 


Raw Meat in the Diarrhœa of Children.—We desire to call the attention of our readers to the excellent effects of raw meat in the colliquative diarrhœa of children, in the hands of Dr. Weisse, of St. Petersbug. Seventeen years ago, Dr. Weisse called the attention of the profession to this subject, and since that time numerous writers have confirmed his views. The meat is reduced to a pulp, by scraping, and given, to the exclusion of all other treatment. Considering the great prevalence of the disease at the present time, and the ease with which the treatment can be adopted, we think it would be well worth while to try the experiment. We would also recall to mind that the same remedy has been found of much efficacy in various diseases of the stomach, accompanied with difficult digestion, in adults as well as in children.—*Boston Medical Journal.*

